# Cytoprotective Activities of Milk Thistle Seed Oil Used in Traditional Tunisian Medicine on 7-Ketocholesterol and 24S-Hydroxycholesterol-Induced Toxicity on 158N Murine Oligodendrocytes

**DOI:** 10.3390/antiox7070095

**Published:** 2018-07-19

**Authors:** Wiem Meddeb, Leila Rezig, Amira Zarrouk, Thomas Nury, Anne Vejux, Michel Prost, Lionel Bretillon, Mondher Mejri, Gérard Lizard

**Affiliations:** 1 Team Bio-PeroxIL, Biochemistry of the Peroxisome, Inflammation and Lipid Metabolism (EA7270)/University Bourgogne Franche-Comté/Inserm, 21000 Dijon, France; wiem.meddeb7@gmail.com (W.M.); thomas.nury@u-bourgogne.fr (T.N.); anne.vejux@u-bourgogne.fr (A.V.); 2 Department of Biotechnology, Institut Préparatoire aux Etudes Scientifiques et Techniques (IPEST), La Marsa 2070, Tunisia; mondhermejri54@gmail.com; 3 Laboratory Matériaux, Molécules et Applications (LMMA), University of Carthage, La Marsa, Tunis 2078, Tunisia; 4 Faculty of Science of Bizerte, Zarzouna, Bizerte 7021, Tunisia; 5 Food Conservation and Valorization Laboratory, High Institute of Food Industries, 58 Avenue Alain Savary, El Khadra City, Tunis 1003, Tunisia; l_rezig@yahoo.fr; 6 Lab. NAFS, Nutrition—Functional Food & Vascular Diseases LR12-ES-05, University of Monastir, Monastir 5000, Tunisia; zarroukamira@gmail.com; 7 Faculty of Medicine, University of Sousse, Sousse 4002, Tunisia; 8 Laboratoire Spiral, 21560 Couternon, France; michelprost.spiral@wanadoo.fr; 9 Eye & Nutrition Research Group, CSGA, UMR 1324 INRA, 6265 CNRS, 21000 Dijon, France; lionel.bretillon@inra.fr; 10 Department of Biotechnology, University of Jendouba, Jendouba 8100, Tunisia

**Keywords:** 158N murine oligodentrocytes, milk thistle seed oil, *Silybum marianum*, 7-ketocholesterol, 24S-hydroxycholesterol, cytoprotection

## Abstract

The Asteraceae family is economically very important, because many of these plants are grown mainly for their food value, such as lettuce (Lactuca), chicory (Cichorium), and sunflower (Heliantus aminus). One of the typical properties of this family, which includes milk thistle (*Sylibum marianum*), is the richness of the oil in various compounds (flavonoids, alkaloids, tocopherols, and unsaturated fatty acids). Currently, and for the coming decades, age-related diseases, including neurodegenerative diseases, are a major public health problem. Preventing their appearance or opposing their evolution is a major objective. In this context, the cytoprotective activities of milk thistle seed oil produced in Tunisia were studied on the 158N model using 7-ketocholesterol (7KC) and 24S-hydroxycholesterol (24S) as cytotoxic agents. 7KC and 24S were used because they can be increased in the brain and body fluids of patients with major age-related neurodegenerative diseases, such as Alzheimer’s and Parkinson’s diseases. In order to evaluate the cytoprotective properties of milk thistle seed oil, complementary techniques of microscopy, flow cytometry, and biochemistry were used. The chemical composition of milk thistle seed oil has also been determined by various chromatography techniques. Milk thistle seed oils from different area of Tunisia are rich in tocopherols and are strongly antioxidant according to various biochemical tests (KRL (Kit Radicaux Libres), FRAP (Ferric Reducing Antioxidant Power), and DPPH (2,2-diphenyl-1-picrylhydrazyl)). The main fatty acids are linoleic acid (C18:2 n-6) and oleic acid (C18:1 n-9). The main polyphenols identified are homovanillic acid, p-coumaric acid, quercetin, and apigenin, with a predominance of vanillic acid. On 158N cells, milk thistle seed oil attenuates the cytotoxicity of 7KC and 24S including: loss of cell adhesion, increased plasma membrane permeability, mitochondrial dysfunction, overproduction of reactive oxygen species, induction of apoptosis, and autophagy. The attenuation of the cytotoxicity of 7KC and 24S observed with the milk thistle seed oil is in the order of that observed with α-tocopherol used as a positive control. In the presence of nigella seed oil, considered potentially cytotoxic, no cytoprotective effects were observed. Given the chemical characteristics, antioxidant properties, and cytoprotective activities of milk thistle seed oil, our results highlight the potential benefit of this oil for human health.

## 1. Introduction

Ageing and age-related diseases, including neurodegenerative diseases, are dependent on genetic and epigenetic factors, including environmental and lifestyle parameters. In nerve cells, these factors can favor neurodegeneration and increase the risk of Alzheimer’s disease and Parkinson disease which are age-related diseases [1]. It is therefore important to determine the main causes of these neurodegenerative diseases and to better understand their physiopathology. Indeed, this will permit further identification of natural or synthetic compounds that are capable of preventing neurodegeneration which has increased in developed and developing countries mainly due to the enhancement of life expectancy [2].

Among the potentially deleterious compounds present in the body, several oxysterols (which are cholesterol derivatives brought by the diet, formed by auto-oxidation and/or enzymatically) play important roles in cell metabolism, RedOx balance and inflammation, particularly in vascular and nerve cells which are strongly affected by the aging process [3,4,5,6]. 

In vitro data have shown that the oxysterols negatively affect many cell types: the brain, eyes, heart, bone, colon, and prostate cells [7]. Oxysterols have been identified as important actors in the pathophysiology of several age-related diseases, such as atherosclerosis, type 2 diabetes mellitus, Alzheimer’s disease, age-related macular degeneration (ARMD) and cataract, osteoporosis, and some forms of cancer (colon carcinoma and prostate cancer) [4,6,7]. 

7-ketocholesterol (7KC), resulting from the auto-oxidation of cholesterol [8], is often present in enhanced levels in the tissues, plasma, and/or cerebrospinal fluid of patients with cardiovascular diseases, ARMD, and neurodegenerative diseases [9,10,11,12,13]. This oxysterol is a powerful inducer of oxidative stress, inflammation, cell death (apoptosis and autophagy (oxiapoptophagy: OXIdative stress + APOPTOsis + autoPHAGY) in different cell lines (monocytes, neurons, oligodendrocytes, retinal pigment epithelial cells). Another important oxysterol is 24S-hydroxycholesterol (24S). This oxysterol, also named cerebrosterol, which is synthesized in the brain by the neurons via the enzyme CYP46A1, is able to cross the blood–brain barrier for further degradation in the liver [11]. 

To prevent oxysterol-induced cytotoxic activities (oxidative stress, inflammation, and cell death) [4], it is important to identify natural and synthetic compounds, and mixture of compounds, that are capable of counteracting or attenuating their side effects.

Natural products are generally classified as primary or secondary metabolites. Among the primary metabolites that are widely distributed in nature are vegetable oils [14]. Several plant extracts are known for their preventive as well as curative effects for age-related diseases, including cardiovascular diseases, neurodegenerative diseases, ocular diseases, intestinal cancers, and cancer of the lungs, prostate and breast.

The extract of pistachio seeds induces a decrease in the proliferation of MCF-7 (Michigan Cancer Foundation–*7*) breast cancer cells and could be exploited as a “natural food additive” in combination with chemotherapy treatment [15]. Other plant extracts have also shown anti-proliferative effects, such as milk thistle seed extract and pumpkin seed extract which inhibit the proliferation of Human Colon Cancer Cells (HT-29) [16]. Work realized by Rahal et al. [17] highlighted the biological activity of milk thistle oil on a colon cancer cell line, Caco-2. These extracts contain bioactive components which could be responsible for their therapeutic effects, such as antioxidants, tocopherols, and carotenoids [16,18]. It has also been shown that black and red raspberry extracts and grapes, which have anti-proliferative activity against human HT-29 cancer cells, contain elevated concentrations of phenols and anthocyanins, tocopherols and carotenoids, and α-linolenic acid (C18:3 n-3) [16,18]. The α-linolenic acid found in these extracts can be converted to eicosapentaenoic acid (EPA: C20:5 n-3) and docosahexaenoic acid (DHA: C22:6 n-3) which can have cytoprotective activity against several chronic human diseases, including cardiovascular diseases and cancer [19,20].

Among the biologically active natural components present in milk thistle (*Silybum marianum*), silymarin is a polyphenol with antioxidant properties that has been reported to have anti-proliferative effects against several cancer cells [21,22]. The anti-proliferative effects of milk thistle seed components have been reported in several cancers: prostate [21,23], bladder [24,25], colon [26,27], lung [21,28], and breast cancer cells [22]; the associated mechanisms include cell cycle arrest, cyclin-dependent kinase and cyclin inhibition, and induction of apoptosis. However, one study on breast carcinogenesis in rats and mice demonstrated an increase in breast tumors with silymarin treatment compared to a control [29]. The use of silymarin as a molecule that can prevent cancer has been summarized by Vejux et al., exposing its effects on the process of carcinogenesis, oxidative stress, cell cycle arrest, kinase pathways, and cell death [30]. 

Work realized by Kittur et al. [31], which evaluated the effects of milk thistle extract on the differentiation and survival of neural cells, demonstrates that this extract can promote differentiation and neuronal survival (evocating neurotrophic activities), and protect the neurons of the rat hippocampus against cell death induced by oxidative stress. These latter data suggest a potential benefit of the chemicals present in milk thistle on the nervous system.

Other plant extracts of *Centella Asiatic*, which is known for its pharmacological effects in the central nervous system [32], have also shown neuroprotective effects, such as the prevention of mitochondrial dysfunction, increased expression of antioxidant enzymes, and decreased lipid peroxidation, which are the main causes of dopaminergic neurodegeneration in Parkinson’s disease.

Altogether, these studies suggest that several plant extracts, including that of milk thistle, could be used to prevent neurodegenerative diseases. Indeed, some of these compounds, especially tocopherols and polyphenols, are capable to cross the blood–brain barrier. Currently, herbal products are increasingly being used as dietary supplements and therapeutic agents. Most of these natural products are used in traditional medicine due to their antioxidant properties. Among the oils that are the most well known for their pharmacological properties in traditional Arab herbal medicine, milk thistle seed oil is very rich in α-tocopherol. As α-tocopherol has been shown to have neuroprotective effects, milk thistle seed oil could be a useful candidate for preventing the cytotoxic effects induced by 7KC and 24S which can be observed at increased levels in the brains and plasma of patients with neurodegenerative diseases [6,11,13]. 

In the present study, the chemical compositions (fatty acids, polyphenols, phytosterols, tocopherols) of milk thistle seed oils from different area of Tunisia (Zaghouan, Bizete, Sousse) were determined as well as their antioxidant properties. The ability of milk thistle oil to attenuate 7KC- and 24S-induced side effects (inhibition of cell proliferation, mitochondrial dysfunction, oxidative stress, apoptosis, and autophagy) was also studied on 158N murine oligodendrocytes. As nigella seed oil is considered potentially cytotoxic based on popular empirical knowledge, the chemical characteristics of this oil were also determined as well as its ability to prevent, or not, 7KC- and 24S-induced side effects on 158N cells. It is noteworthy that, whereas no cytoprotective effects were observed with nigella seed oil, a marked attenuation of 7KC- and 24S-induced side effects was observed with milk thistle seed oil from different areas of Tunisia (Zaghouan, Bizerte, and Sousse).

## 2. Materials and Methods

### 2.1. Cold Extraction of Oils

#### 2.1.1. Seed Materials

The present study mainly concerns milk thistle (*Silybum marianum*) seeds originating from three different area of Tunisia, namely Zaghouan (36°24′ N 10°09′ E), Bizerte (37°16′28′′ N 9°52′26′′ E), and Sousse (35°50′ N 10°38′ E). The plants were harvested in May and June 2014, during which time the plants reach maturity and senescence. Currently, as little is known about the effects of the environment (soil, climate) on the characteristics of milk thistle seed oils, the seed were collected in three different area of Tunisia.

Seeds of *Nigella sativa* (Asian origin, also named black cumin) were also used; they were bought from souk Blat (Tunisia).

#### 2.1.2. Extraction of Oils

The seeds of milk thistle (*Silybum marianum* L.) and nigella (*Nigella sativa*) were obtained by cold pressing. This technique provides a cold extraction of the solid sample oil contained in a plant hopper. The oils obtained were stored at (−20 °C) for further analyses.

### 2.2. Cell Culture and Treatments

Murine oligodendrocytes (158N) [33] were cultured in Dulbecco’s Modified Eagle’s Medium (DMEM) (Lonza, Amboise, France) with 5% (*v*/*v*) heat-inactivated fetal bovine serum (FBS) (Dutscher, Brumath, France) and 1% antibiotics (penicillin, streptomycin) (Dutscher). The cells were inoculated at 30,000 cells/cm^2^, either in petri dishes (100 × 20 mm, FALCON, Corning, NY, USA) with 10 mL of culture medium, or at 240,000 cells per well on six-well plates with 2 mL of culture medium.

7-Ketocholesterol (7KC; Ref: C2394) was from Sigma-Aldrich (St Quentin Fallavier, France) and 24S-hydroxycholesterol (24S) was provided by Mohammad Samadi (University of Lorraine, Metz, France). The stock solutions of 7KC and 24S were prepared at 800 μg/mL (2 mM), as described by Ragot et al. [34]. After 24 h of culture, treatments with 7KC and 24S (25 and 50 µM) were performed for 24 h with or without milk thistle seed oil. *Nigella sativa* seed oil, known for its potential cytotoxic effects, was simultaneously used, as well as α-tocopherol (Sigma-Aldrich: 400 μM) which is known to prevent 7KC-induced side effects [34]. The concentrations of 7KC, 24S, and α-tocopherol as well as the times of treatment were in accordance with those described with these compounds in previous studies [35,36,37,38,39,40,41].

Stock solutions of milk thistle seed oils (milk thistle oils from different area of Tunisia: Zaghouan, Bizerte, and Sousse) and nigella seed oil were prepared at 10% (*v*/*v*) in absolute ethanol. For cell processing, one volume of this stock solution was diluted into 100 volumes of culture medium; the final oil concentration was 0.1% (*v*/*v*). 

When the cells were treated with 7KC and 24S, associated with milk thistle seed oil, nigella seed oil, or α-tocopherol, these compounds were added 2 h before 7KC and 24S. Based on the concentrations of 7KC and 24S used, it was assumed that the intracellular oxysterol content obtained in vitro was in the range of those that occur in vivo [34,42]. α-Tocopherol was used at the highest non-cytotoxic concentration (400 μM) capable of preventing 7KC and 24S-induced oxiapoptophagy in many cell types [35,36,38,39,43]. 

### 2.3. Determination of the Fatty Acid Profiles of Milk Thistle Oils and Nigella Oil by Gas Chromatography

The lipids were extracted from the various oils (milk thistle seed oils from different areas of Tunisia (Zaghouan, Bizerte, Sousse) and nigella oil) according to the method of Moilanen and Nikkari [44]. C19:0 was used as an internal standard. The lipids were transmethylated using boron trifluoride in methanol, in accordance with Morrison & Smith [45]. The fatty acid methyl esters were then analyzed under the same conditions as those described by Debbabi et al. [46] and Badreddine et al. [47]. The data were processed with EZChrom Elite software (Agilent Technologies, Massy, France) and are reported in mg/g total lipids.

### 2.4. Determination of the Tocopherol Profile of Milk Thistle Oils and Nigella Oil by High Pressure Liquid Chromatography

Before the high performance liquid chromatography (HPLC) analysis, 0.5 g of the seed oil was diluted with 5 mL of hexane, and 5 μL of the sample was then injected. The tocopherol composition of the seed oils was determined by HPLC in accordance with the standard [NF EN ISO 9936] [46,47]. The samples were then analyzed by HPLC (Agilent 1100, Palo Alto, CA, USA), as previously described by Debbabi et al. [46] and Badreddine et al. [47]. The quantification was based on an external standard method and the tocopherol standards were mixed in a solution of hexane (2 mg/mL) prepared on the basis of the standard compounds: α-, β-, γ- and δ-tocopherols.

### 2.5. Analysis of Polyphenols of Milk Thistle Oils and Nigella Oil

Three grams of each oil were dissolved in 6 mL of petroleum ether and then, were cleaned on a cartridge of silica for solide phase extraction (SPE) (conditioned with 6 mL of petroleum ether). The cartridge was then washed with 12 mL of petroleum ether and dried under a stream of nitrogen for 10 min. The polyphenolic compounds were eluted with 8 mL of methanol/distilled water 80/20 (*v*/*v*) and then with 8 mL of acetonitrile. The eluate was evaporated under reduced pressure at 50 °C. The residue was taken up in 1 mL of a mixture of methanol/distilled water 60/40 (*v*/*v*). The resulting extract was filtered through an 0.45 μm nylon membrane. The analysis of the polyphenols was done by the HPLC-MS method, as described by Badreddine et al. [47]. The phenolic compounds were identified using a combination of high performance liquid chromatography (HPLC) with Agilent 1100 diode array detection and electrospray ionization mass spectrometry (ESI-LC-MS) based on their ultraviolet (UV) spectra, comparing the spectra with those of the authentic standards available.

### 2.6. Analysis of Phytosterols of Milk Thistle Oils and Nigella Oil

The analysis of sterols was realized as described by Badreddine et al. [47], Mounts et al. [48], and Park et al. [49]. Quantitation of phytosterols and phytostanols was performed by adding cholestanol as an internal standard. The analyses were performed in triplicate.

### 2.7. KRL (Kit Radicaux Libres) Test

The antioxidant potential of milk thistle oils, nigella oil, α-tocopherol, silymarin, resveratrol, and ferrulic acid was evaluated with the KRL (Kit Radicaux Libres) test, as previously described [46,50]. The data are presented in Trolox equivalents. Hemolysis was recorded using a 96-well microplate reader by measuring the decay of the optical density at 450 nm on a spectrophotometer. For each well, absorbance measurements were made 75 times, once every 150 s.

### 2.8. Determination of Ferric Reducing Antioxidant Power (FRAP)

The Ferric Reducing Antioxidant Power (FRAP) test, which measures the antioxidant potential of compounds or mixtures of compounds by reducing ferric iron (Fe^3+^) to ferrous iron (Fe^2+^), was realized as previously described [51,52]. The absorbance of the reaction medium was read at 700 nm against a blank prepared by replacing the extract with distilled water to calibrate the spectrophotometer. An increase in absorbance corresponded to an increase in the reducing power of the extracts tested [53]. The reducing power of the extracts in mg of Trolox per g of dry matter (mg of Trolox/g of dry matter) was calculated as follows:

Iron reduction power (%) = [Absorbance control 700 − Absorbance sample 700)/Absorbance control 700] × 100.

For milk thistle oils, nigella oil, α-tocopherol, ferrulic acid, resveratrol, and silymarin (used as a positive control), the FRAP method was performed as described by Debbabi et al. [46]. For the various oils analyzed, the corresponding antioxidant power was expressed in Trolox equivalents for 1 mL of oil.

### 2.9. DPPH Assay

The DPPH assay for 2,2-diphenyl-1-picrylhydrazyl (C18H12N5O6) (Sigma-Aldrich) can be regarded as a free radical scavenger test. The DPPH assay is used to measure the anti-radical activity of compounds or mixtures of compounds. The absorbance was read at 517 nm (Sunrise spectrophotometer, Tecan, Lyon, France). The negative control contained 100 μL of water and 100 μL of the DPPH solution. The antioxidant potentials of α-tocopherol, silymarin, ferrulic acid, and resveratrol (used as positive controls) were estimated in Trolox equivalents (1 mole of α-tocopherol, silymarin, ferrulic acid, or resveratrol is equivalent to X mole of Trolox; the calibration curve was obtained with Trolox) [46,47]. For the milk thistle oils from the different areas of Tunisia and for the nigella oil, the corresponding antioxidant powers were expressed as the Trolox equivalent for 1 mL of oil.

### 2.10. Crystal Violet Test

Crystal violet staining (Sigma-Aldrich) is used to highlight cell proliferation by estimating the proportion of adherent cells [54]. After cell staining with violet crystal (Sigma-Aldrich) in 12-well plates, the absorbance was measured at 598 nm with a microplate reader (Sunrise spectrophotometer, Tecan, Lyon, France).

### 2.11. Measurement of Mitochondrial Activity with the MTT (Methylthiazolyldiphenyl-Tetrazolium Bromide) Test

To evaluate the mitochondrial activity, the MTT test was used. It is based on the reduction of MTT salt to formazan in metabolically active cells [55,56]. At the end of the treatment, the cells were incubated in the presence of MTT for 3 h, formazan was solubilized by the addition of dimethylsulfoxide (Sigma-Aldrich), and the optical density was measured at 570 nm with a microplate reader (Sunrise spectrophotometer).

### 2.12. Measurement of Superoxide Anions Production with Dihydroethidium

Dihydroethidium (DHE, Life Technologies) was used to detect overproduction of the superoxide anion (O_2_•^−^) [57]. DHE (2 µM final concentration, incubation: 15 min, 37 °C) is rapidly oxidized in the cells in ethidium by reactive oxygen species, mainly O_2_•^−^ [58]. The fluorescent signals were collected through a 590/20 nm bandpass filter on a logarithmic scale on a GALAXY flow cytometer (Partec); 10,000 cells were acquired. Flomax (Partec) or Flowjo (Tree Star Inc., Ashland, OR, USA) was used to analyze the data.

### 2.13. Measurement of Plasma Membrane Permeability with Propidium Iodide

The measurement of plasma membrane permeability was based on the property of propidium iodide (PI) to enter in dead cells and/or in cells with damaged plasma membranes [59]. The fluorescence of PI (used at 1 µg/mL) was collected with a 630 nm pass filter on a Galaxy flow cytometer (Partec). Ten thousand cells were acquired, and Flomax (Partec) or Flowjo (Tree Star Inc.) was used to analyze the data. 

### 2.14. Quantification of Apoptotic Cells after Nuclei Staining with Hoechst 33342

The nuclear morphology of apoptotic cells can be determined after staining with Hoechst 33342 (2 µg/mL). Observations were performed by fluorescence microscopy under a UV light with an Axioskop microscop (Zeiss, Jena, Germany). The percentages of cells with condensed and/or fragmented nuclei, which are characteristic of apoptotic cells, were determined [59]. For each sample, 300 cells were observed. The cells were deposited on glass slides by cytocentrifugation (5 min, 1500 rpm) with a cytospin 2 (Shandon, Cheshire, UK).

### 2.15. Analysis of Caspase-3 and Microtubule-Associated Protein 1 Light Chain 3 (LC3) by Polyacrylamide Gel Electrophoresis and Western Blotb

The analysis of caspase-3 and LC3 was realized by polyacrylamide gel electrophoresis and Western blot analysis, as previously described by Nury et al. [39]. Briefly, cell lysis was performed in a Radioimmunoprecipitation assay buffer (RIPA buffer) containing protease inhibitors (Roche Diagnostics, Indianapolis, IN, USA) for 30 min on ice followed by centrifugation (20 min, 20,000× *g*) to remove cell debris. Seventy micrograms of protein were separated on a 14% SDS-PAGE gel for caspase-3 and LC3 and transferred to a nitrocellulose membrane (Bio-Rad, Marne La Coquette, France). After blocking nonspecific binding sites for 2 h with 5% milk powder in PBST (PBS, 0.1% Tween 20, pH 7.2), the membrane was incubated overnight at 4 °C with the primary antibody diluted in PBST—antibodies to caspase-3 (rabbit polyclonal antibody, Ozyme/Cell Signaling, ref: # 9662) and LC3-I/II (rabbit polyclonal antibody, Sigma-Aldrich, ref.: L8918)—at a final dilution of 1/1000. To standardize the results, an antibody directed against β-actin (mouse monoclonal antibody, ref: A2228, Sigma-Aldrich) was used at a final concentration of 1/10,000. After 2 washings with PBST, the membrane was incubated for 1 h at room temperature with a goat anti-rabbit antibody conjugated with horseradish peroxidase (Cell Signaling, No. 7074) diluted to 1/5000. The membrane was then revealed using an improved chemiluminescence detection kit (Supersignal West Femto Maximum Sensitivity Substrate, Thermo-Scientific) and ChemiDoc^TM^ XRS+ (Bio-Rad). The (LC3-II/LC3-I) ratio was calculated and the levels of cleaved caspase-3 were normalized with actin with Image Lab software (Bio-Rad). 

### 2.16. Statistical Analyses

The data are expressed as means ± standard deviations (SDs). Statistical analyses involved two-way ANOVAs (Tukey Multiple Comparison Test) with GraphPad Prism 6.07 software (GraphPad Software, La Jolla, CA, USA). *p*-Values less than or equal to 0.05 were considered statistically significant.

## 3. Results and Discussion

### 3.1. Profiles of Fatty Acids, Phytosterols, Tocopherols, and Polyphenols Cold-Extracted from Milk Thistle Seed Oils from Different Area of Tunisia

Milk thistle seed oil (MTSO) is known for its high level of secondary metabolites, such as phenolic acids, tocopherols, fatty acids, and phytosterols, which depend on many intrinsic (genetic) and extrinsic (environmental) parameters [60,61,62]. In the present study, we focused on the fatty acids, phytosterols, polyphenols and tocopherols present in MTSO. Data obtained were compared with nigella seed oil which is mainly empirically known for its toxic activity.

Milk thistle seed oil (MTSO; from Tunisia—Bizerte, Sousse and Zaghouan) and nigella seed oil (NSO; from Tunisia) were analyzed. The fatty acid compositions of these oils are given in Table 1. Linoleic acid (C18:2 n-6) was the most abundant polyunsaturated fatty acid (PUFA) detected in MTSO and NSO. The results show that the oils used in this experiment had high levels of linoleic acid. From a nutritional point of view, as for many other dietary oils, MTSO is a good source of essential fatty acids, especially linoleic acid. Another unsaturated fatty acid quantitatively high in MTSO was oleic acid (C18:1 n-9). Palmitic acid (C16:0) was the most abundant saturated fatty acid in MTSO and NSO, followed by stearic acid (C18:0). Very long-chain saturated fatty acids (C22:0 and C24:0) were found to have high amounts in MTSO. In addition to the fatty acid profiles, the composition of tocopherols is an important parameter to define the potential health benefits of vegetable oils. The tocopherol contents of the oils studied are given in Table 2. The results show that α- and β-tocopherols were the only ones detected in cold-pressed MTSO from Bizerte and represented 47.65 mg/kg and 1.91 mg/kg, respectively. In the oils from Zaghouan and Sousse, the four tocopherols were detected with a predominance of α-tocopherol which represented 98.92% and 88.59%, respectively. The amounts of α-tocopherol in the MTSO of Zaghouan and Sousse were in the same range as those reported by El-Mallah et al. [60] in MTSO extracted with chloroform-methanol (84.5%) and those reported by Fathi-Achachlouei and Azadmard-Damirchi [61] for Iranian varieties of Barak, and for MTSO extracted by hexane/isopropanol (84.9% and 86.36%, respectively). The amount of α-tocopherol was greater than that reported by Parry et al. [16] (78.78%) in cold-pressed MTSO, and by Hasanlou et al. [63] in MTSO (69.02%) extracted by petroleum ether (Soxhlet apparatus). In the MTSO from Sousse, the high content of α-tocopherol (considered to be the most biologically active tocopherol) [64] was followed by γ-tocopherol, β-tocopherol, and δ-tocopherol. The amounts of β- and γ-tocopherol were greater than those observed in the MTSO from Zaghouan. Thus, the cold pressed MTSO from Zaghouan is an ideal source of total α-tocopherol, while that of Sousse could serve as a source of γ-tocopherol. It is noteworthy that compared to olive and argan oils, the highest values of α-tocopherol were observed in MTSO [46,47].

As phytosterols are components that are present in the unsaponifiable lipid fraction of plant cell membranes [64] and are abundant in vegetable oils (nuts, cereals), it was important to determine the content and composition of the sterols in the MTSO studied (Table 3). β-Sitosterol was the main sterol in all oils. Schottenol was also detected at a high level in MTSO and, in particular, in Zaghouan MTSO. Campesterol was determined in appreciable amounts, especially in the MTSO from Sousse. The presence of cholesterol is in agreement with the results reported by Fathi-Achachlouei and Azadmard-Damirchi [61] as well as by Dabbour et al. [62]. Cholesterol could be formed by demethylation of sitosterol. The synthesis of cholesterol in plants remains to be clarified [65]. Cholesterol can be localized in the nucleus, chloroplasts, and microsomes in free or esterified form. Cholesterol is an important constituent of the leaf wall. It should be noted that high levels of cholesterol have been reported in palm, corn, and sunflower oils [66]. Cholesterol has never been identified, even at very low levels, in olive and argan oils, whatever the geographic origin of the oils [46,47]. 

The polyphenol compositions of the oils are given in Table 4. Homovanillic acid, vanillin, p-coumaric acid, quercetin-3β-glucoside, quercetin, and apigenin were identified in MTSO. In nigella seed oil, 2,6-dihydroxybenzoic acid, homovanillic thymoquinone acid, vanillin and some amounts of chlorogenic acid, ferulic acid, quercetin, and apigenin were detected.

As many compounds present in MTSO (phytosterols, polyphenols, tocopherols) are able to prevent nerve cell dysfunction and can cross the blood–brain barrier [67,68], these data reinforced our interest in evaluating the ability of MTSO to prevent the toxic effects of 7KC and 24S [68,69]. So, the ability of MTSO to prevent the side effects induced by 7KC and 24S was compared to α-tocopherol (used as a positive cytoprotective control) and to nigella seed oil (supposed as potentially cytotoxic).

### 3.2. Evaluation of the Antioxidant Properties of Milk Thistle Seed Oil from Different Area of Tunisia with the KRL, FRAP, and DPPH Tests

The antioxidant properties of α-tocopherol, silymarin, resveratrol, ferrulic acid, and milk thistle seed oil from different area of Tunisia (Zaghouan, Bizerte and Sousse) were determined by three complementary methods: the KRL (Kit Radicaux Libres), the Ferric Reducing Antioxidant Power (FRAP) test, and the DPPH test. These three tests provide similar information for the different compounds studied (Table 5). The α-tocopherol data were in the range of those previously reported and allowed us to validate our data [46,47]. According to the three tests, the antioxidant properties of MTSO and nigella seed oil were similar.

### 3.3. Effects of Milk Thistle Seed Oil on 7-Ketocholesterol- and 24S-Hydroxycholesterol-Induced Mitochondrial Dysfunction and Cell Growth Inhibition, Assessed with the MTT and Crystal Violet Tests 

**Figure 1:**
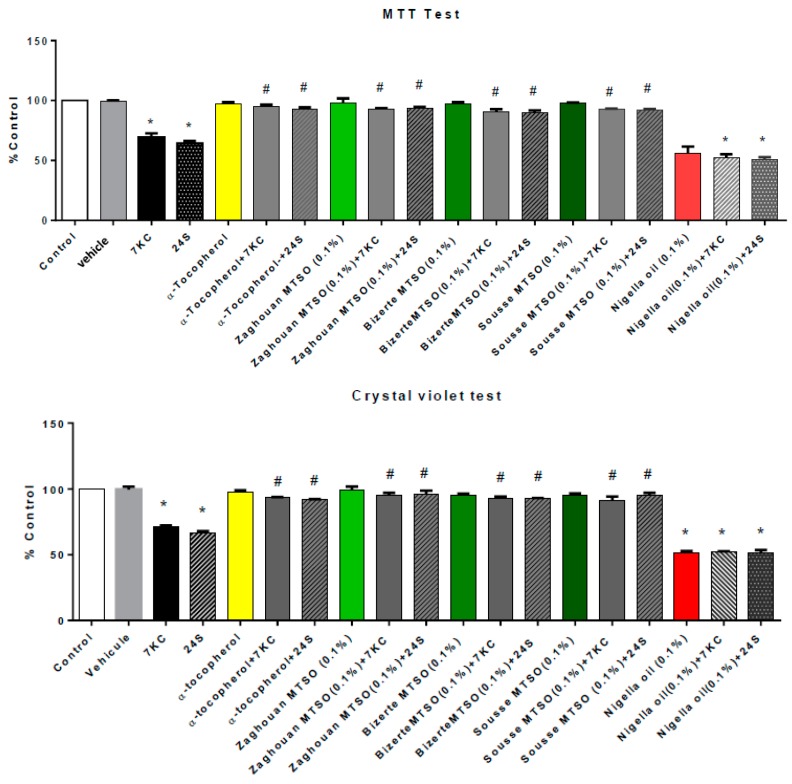
Evaluation of the effects of milk thistle seed oil (MTSO) on 7-ketocholesterol (7KC)- and 24S-hydroxycholesterol (24S)-induced mitochondrial dysfunction and cell growth inhibition in 158N murine oligodendrocytes with the MTT (Methylthiazolyldiphenyl-tetrazolium bromide) and crystal violet tests. After 24 h of culture, 158N murine oligodendrocytes were cultured for 24 h without or with 7KC or 24S (25 µM) in the absence or presence of milk thistle seed oils (MTSO; area: Zaghouan, Bizerte, and Sousse; 0.1% *v*/*v*) or α-tocopherol (400 µM), which was used as a positive control. MTSO and nigella seed oil (0.1% *v*/*v*), and α-tocopherol were added to the culture medium 2 h before 7KC and 24S. The cytoprotective effects of MTSO on 7KC- and 24S-induced mitochondrial dysfunction (decreased activity of succinate dehydrogenase, a mitochondrial enzyme belonging to the Krebs cycle) and inhibition of cell growth were evaluated with the MTT and crystal violet tests, respectively. Data are the means ± standard deviations (SDs) of two independent experiments carried out in triplicate. The significance of the difference between vehicle and cells treated with 7KC, 24S, MTSO and nigella seed oil or α-tocopherol was calculated with ANOVA tests (Sidak’s multiple comparisons); * *p* ≤ 0.05. The significance of the difference between cells treated with 7KC alone, 7KC or 24S with MTSO, or 7KC and 24S with α-tocopherol was calculated by ANOVA tests (Sidak’s multiple comparisons); # *p* ≤ 0.05. No significant differences were found between control and vehicle-treated cells (ethanol).

To determine the impact of MTSO (0.1% *v*/*v*, final concentration) on the loss of mitochondrial activity and on cell growth inhibition triggered by 7KC and 24S (25 μM, 24 h), the MTT and crystal violet tests were used, respectively. With the MTT test, measurement of the enzymatic activity of succinate dehydrogenase (a mitochondrial enzyme of the Krebs cycle) showed lower MTT values following 7KC and 24S treatment, supporting a loss of ΔΨm and/or a reduced cell growth and/or a loss of cell adhesion [70,71,72] (Figure 1). The crystal violet test, which permits the evaluation of cell growth, also showed a significant decrease in 158N adherent cells and/or a loss of cell adhesion after treatment with 7KC and 24S for 24 h (Figure 1). In the presence of MTSO or α-tocopherol (400 μM), which was used as a positive control [34], the toxic effects of 7KC (lower MTT values, lower crystal violet values) were significantly reduced, whereas no cytoprotective effects of nigella seed oil were found (Figure 1). No significant differences were observed between the controls, vehicle (ethanol)-treated cells, MTSO, and α-tocopherol for cell growth evaluated with the crystal violet test (Figure 1). However, lower MTT and crystal violet values, similar to those observed with 7KC and 24S, were observed with nigella seed oil, and no cytoprotective effects of nigella seed oil were found; nigella seed oil did not prevent 7KC- and 24S-induced mitochondrial dysfunction and cell growth inhibition.

### 3.4. Effects of Milk Thistle Seed Oil on 7-Ketocholesterol and 24S-Hydroxycholesterol-Induced Overproduction of Reactive Oxygen Species

**Figure 2:**
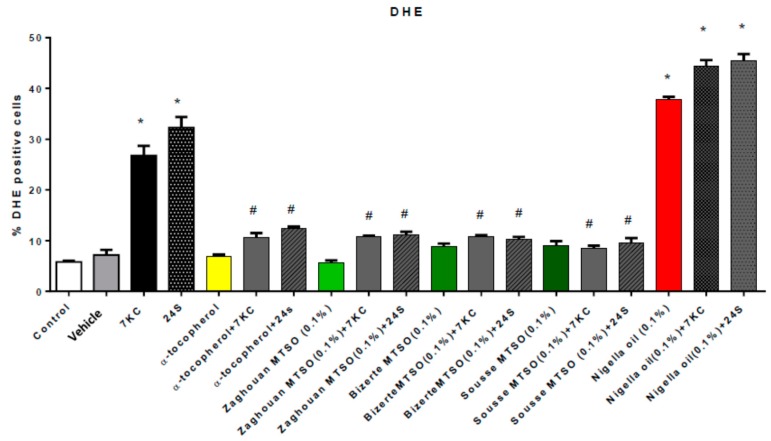
Evaluation of the effect of milk thistle seed oil (MTSO) on 7-ketocholesterol- (7KC) and 24S-hydroxycholesterol (24S)-induced overproduction of reactive oxygen species (ROS) in 158N murine oligodendrocytes. After 24 h of culture, 158N murine oligodendrocytes were cultured for 24 h without or with 7KC (25 µM) or 24S (25 µM) in the absence or presence of MTSO (Area: Zaghouan, Bizerte, and Sousse; 0.1% *v*/*v*) or α-tocopherol (400 µM), which was used as the positive control. MTSO, nigella seed oil, and α-tocopherol were added to the culture medium 2 h before 7KC and 24S. The cytoprotective effect of MTSO on 7KC- and 24S-induced ROS overproduction, mainly superoxide anions, was evaluated by flow cytometry after staining with dihydroethidine (DHE). ROS overproduction was determined by the percentage of dihydroethidine (DHE)-positive cells. Data are means ± SDs of two independent experiments carried out in triplicate. The significance of the differences between vehicle and cells treated with 7KC, MTSO, nigella seed oil, or α-tocopherol was calculated by ANOVA tests (Sidak’s multiple comparisons); * *p* ≤ 0.05. The significance of the differences between cells treated with 7KC or 24S, 7KC or 24S with MTSO, and 7KC or 24S with α-tocopherol was calculated by ANOVA tests (Sidak’s multiple comparisons); # *p* ≤ 0.05. No significant differences between control, vehicle (ethanol), MTSO, and α-tocopherol were observed.

As 7KC induces oxidative stress, which can contribute to oxiapoptophagy, in numerous cell types (murine microglial BV-2 cells [35], human SK-N-BE neuroblastoma cells [72], human U937 monocytic cells [73], and murine oligodendrocyte 158N cells [39,74]), the ability of MTSO to prevent oxidative stress was studied. The effects of MTSO (0.1% *v*/*v*, final concentration) and α-tocopherol (400 μM) on reactive oxygen species (ROS) overproduction under treatment with 7KC and 24S (25 μM, 24 h) were evaluated by flow cytometry after staining with dihydroethidium (DHE). Under treatment with 7KC and 24S, an overproduction of ROS (compared to vehicle-treated cells) was observed. Thus, after treatment with 7KC and 24S, the percentage of DHE-positive cells significantly increased. Treatment with MTSO or α-tocopherol alone had no effect on the production of ROS. When MTSO and α-tocopherol were added with 7KC and 24S, the overproduction of ROS was greatly and significantly attenuated compared to the condition with 7KC and 24S alone (Figure 2). No significant differences were observed between untreated cells, vehicle (ethanol)-treated cells, and MTSO- or α-tocopherol-treated cells (Figure 2). However, an enhancement of ROS overproduction (similar to with 7KC and 24S) was observed with nigella seed oil, and no cytoprotective effects of nigella seed oil were found; nigella seed oil did not prevent 7KC- and 24S-induced ROS overproduction.

### 3.5. Effects of Milk Thistle Seed Oil from Different Areas of Tunisia on Plasma Membrane Permeability under Treatment with 7-Ketocholesterol and 24S-Hydroxycholesterol

**Figure 3:**
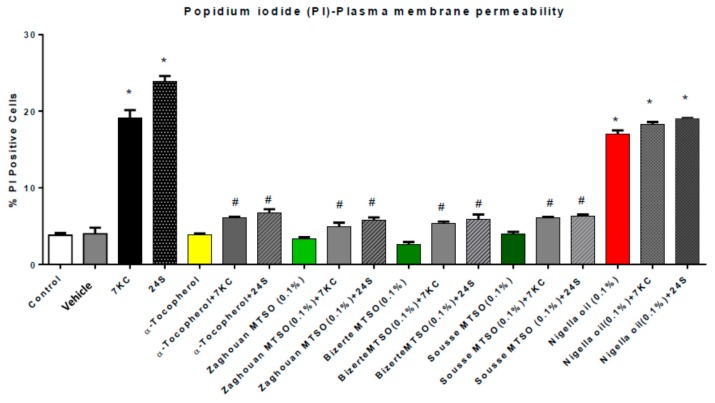
Effect of milk thistle seed oil (MTSO) on 7-ketocholesterol (7KC)- and 24S-hydroxycholesterol (24S)-induced plasma membrane permeability in 158N murine oligodendrocytes. After 24 h of culture, 158N murine oligodendrocytes were cultured for 24 h without or with 7KC or 24S (25 µM) in the absence or presence of MTSO (Area: Zaghouane, Bizerte, and Sousse; 0.1% *v*/*v*) or α-tocopherol (400 µM), which was used as a positive control. MTSO, nigella seed oil, and α-tocopherol were added to the culture medium 2 h before 7KC. The cytoprotective effects of MTSO and nigella seed oil on 7KC and 24S-induced plasma membrane permeability were evaluated by flow cytometry after staining with propidium iodide (PI). Plasma membrane permeability was determined by the percentage of PI positive cells. Data are means ± SDs of two independent experiments carried out in triplicate. The significance of the differences between the vehicle and cells treated with 7KC, MTSO, nigella seed oil, or α-tocopherol was calculated by ANOVA tests (Sidak’s multiple comparisons); * *p* ≤ 0.05. The significance of the differences between the cells treated with 7KC or 24S, 7KC or 24S with MTSO, and 7KC or 24S with α-tocopherol was calculated by ANOVA tests (Sidak’s multiple comparisons); # *p* ≤ 0.05. No significant difference between the control, vehicle (ethanol), MTSO, and α-tocopherol were observed.

The effects of milk thistle oils (0.1% *v*/*v*, final concentration) and α-tocopherol (400 μM) on the plasma membrane alterations induced by 7KC and 24S (25 μM, 24 h) were evaluated by flow cytometry with propidium iodide (PI). In 158N cells, 7KC and 24S induce an increase in plasma membrane permeability (evaluated by the percentage of PI-positive cells). Compared to control cells and vehicle (ethanol)-treated cells, the percentage of PI-positive cells under treatment with 7KC and 24S significantly increased. When MTSO and α-tocopherol were added with 7KC and 24S, the increase in plasma membrane permeability was strongly attenuated (Figure 3). Treatment with MTSO or α-tocopherol had no effect on the plasma membrane permeability. No significant differences between control, vehicle (ethanol), MTSO, and α-tocopherol were observed. (Figure 3). However, an enhancement of plasma membrane permeability (similar than with 7KC and 24S) was observed with nigella seed oil, and no cytoprotective effects of nigella seed oil were found with oxysterol-induced enhancement of membrane permeability.

### 3.6. Effects of Milk Thistle Seed Oil on 7-Ketocholesterol- and 24S-Hydroxycholesterol-Induced Apoptosis and Autophagy

**Figure 4:**
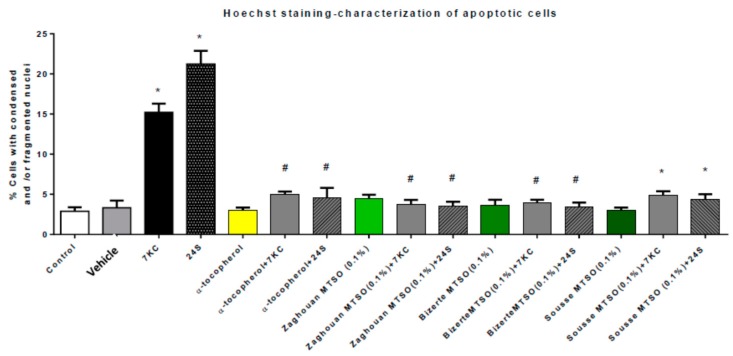
Effects of milk thistle seed oil (MTSO) on 7-ketocholesterol (7KC)- and 24S-hydroxycholesterol (24S)-induced apoptosis evaluated by condensation and/or fragmentation of the nuclei. After 24 h of culture, 158N murine oligodendrocytes were cultured for 24 h with or without 7KC (25 µM) in the absence or presence of MTSO (Area: Zaghouan, Bizerte, and Sousse; 0.1% *v*/*v*) or α-tocopherol (400 µM), which was used as the positive control. MTSO and α-tocopherol were added to the culture medium 2 h before 7KC and 24S. Apoptosis was evaluated by the percentage of apoptotic cells characterized by condensed and/or fragmented nuclei, whereas control cells (untreated cells) had round and regular nuclei. Data are the means ± SDs of two independent experiments carried out in triplicate. The significance of the differences between the vehicle and cells treated with 7KC, MTSO, nigella seed oil, or α-tocopherol was calculated by ANOVA tests (Sidak’s multiple comparisons); * *p* ≤ 0.05. The significance of the differences between cells treated with 7KC or 24S, 7KC or 24S with MTSO, 7KC or 24S with α-tocopherol was calculated by ANOVA tests (Sidak’s multiple comparisons); # *p* ≤ 0.05. No significant differences between the control, vehicle (ethanol), MTSO, and α-tocopherol were observed.

The effects of MTSO (0.1% *v*/*v*) and α-tocopherol (400 μM) on 7KC- and 24S- (25 and/or 50 μM, 24 h) induced apoptosis and autophagy were evaluated by nuclear staining with Hoechst 33342, allowing the identification of cells with apoptotic nuclei (Supplementary Figure S1) by the activation of caspase-3 for apoptosis and by conversion of LC3-I in LC3-II leading to an increase in [LC3-II/LC3-I] for autophagy [74]. The MTSO from Zaghouan was chosen to determine the impact on caspase-3 activation and on the conversion of LC3-I in LC3-II, based on this oil having the highest content of α-tocopherol. As shown by staining with Hoechst 33342, in the presence of 7KC and 24S (25 and/or 50 μM; 24 h), both MTSO (0.1% *v*/*v*) and α-tocopherol (400 μM) were able to reduce apoptosis (Figure 4). 

**Figure 5:**
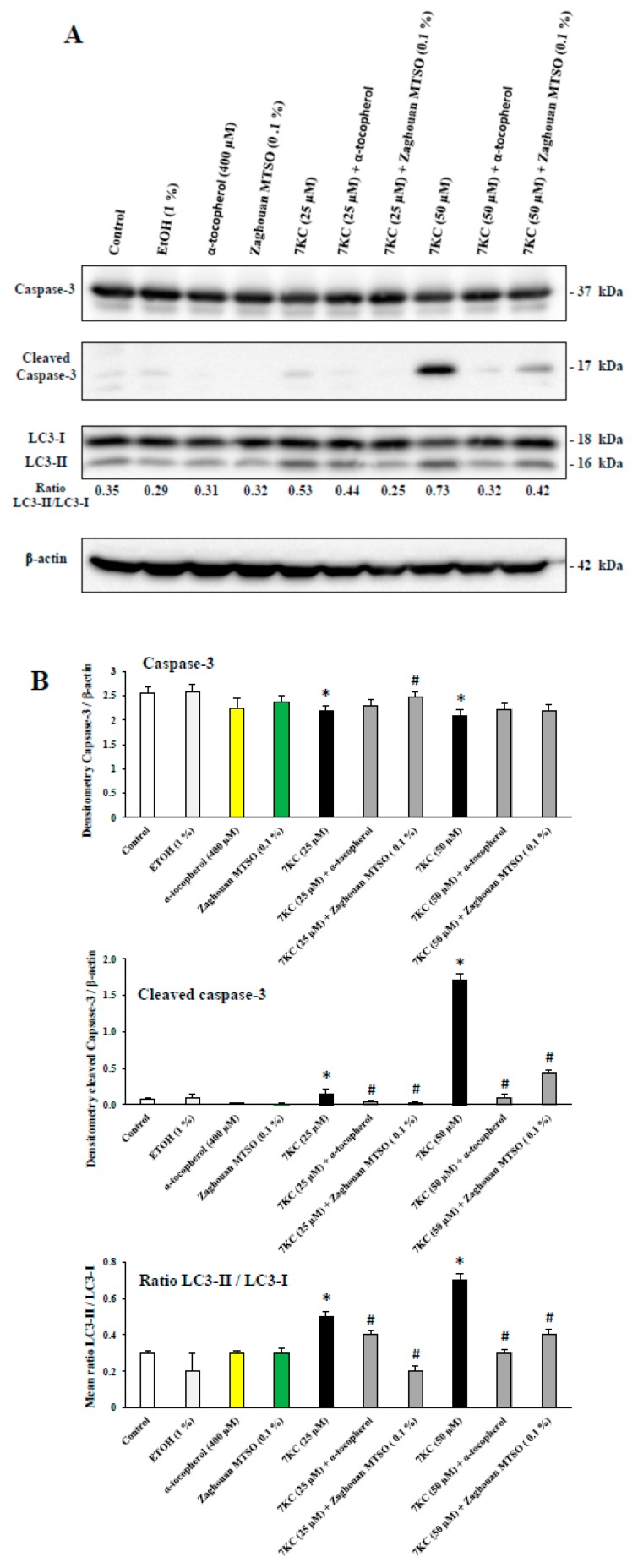
Effects of milk thistle seed oil (MTSO from Zaghouan area, Tunisia) on 7-ketocholesterol (7KC)-induced caspase-3 cleavage and activation of LC3-I in LC3-II. (**A**) After 24 h of culture, 158N murine oligodendrocytes were cultured for 24 h without or with 7KC (25 µM or 50 µM) in the absence or presence of MTSO (Zaghouan; 0.1% *v*/*v*) or α-tocopherol (400 µM), which was used as positive control. MTSO and α-tocopherol were added to the culture medium 2 h before 7KC. Apoptosis was evaluated by caspase-3 activation (cleaved caspase-3) and autophagy by conversion of LC3-I to LC3-II (increased LC3-II/LC3-I ratio). (**B**) The means ± SDs of the densitometric values of 3 independent experiments are shown. The ethanol (EtOH) value corresponds to the highest final EtOH concentration present in the culture medium with 7KC (50 µM) associated with MTSO. The significance of the differences between vehicle and cells treated with 7KC, MTSO or α-tocopherol was calculated by ANOVA tests (Sidak’s multiple comparisons); * *p* ≤ 0.05. The significance of the differences between the cells treated with 7KC, 7KC with MTSO, and 7KC with α-tocopherol was calculated by ANOVA tests (Sidak’s multiple comparisons); # *p* ≤ 0.05. No significant differences between control, vehicle (ethanol)-treated cells, MTSO, and α-tocopherol were observed. No differences were observed between the control, vehicle (ethanol), MTSO, and α-tocopherol.

As shown by Western blotting, in the presence of 7KC (25–50 μM; 24 h), both MTSO (0.1% *v*/*v*) and α-tocopherol (400 μM) were able to prevent (i) apoptosis, revealed by the presence of cleaved caspase-3, and (ii) autophagy, revealed by activation of LC3-I in LC3-II. In the presence of 7KC (25–50 μM, 24 h), caspase-3 was cleaved in a concentration dependent manner (Figure 5); similarly, the [LC3-II/LC3-I] ratio was increased in a concentration dependent manner (Figure 5). The cleavage of caspase-3 and the activation of LC3-I in LC3-II (revealed by an increase in the LC3-II/LC3-I ratio) were attenuated by MTSO from Zaghouan (Figure 5). When the 158N cells were treated with the vehicle (ethanol), MTSO from Zaghouan (0.1% *v*/*v*) or α-tocopherol (400 μM), no caspase-3 cleavage and no increase of the ratio [LC3-II/LC3-I] were observed (Figure 5).

### 3.7. Discussion

The prevalence of some diseases or syndromes increase with age, such as atherosclerosis, Alzheimer’s disease, age-related macular degeneration, cataract, and osteoporosis. All of these diseases involve oxidative stress, inflammation, and/or cell death processes [11].

Oxysterols could play an important role in these different age-related pathologies, in particular, in the pathophysiology of the brain. Indeed, several oxysterols can induce several side effects contributing to neurodegeneration [11].

The most widely-considered oxysterols potentially involved in the pathogenesis of the processes of neurodegenerative diseases are 24S [75], which is of enzymatic origin, and 7KC resulting from cholesterol auto-oxidation [8,76].

In 158N murine oligodendrocytes, 7KC and 24S trigger a complex mode of cell death defined as oxiapoptophagy (OXIdation + APOPTOsis + autoPHAGY), simultaneously involving oxidative stress, apoptosis, and autophagy [73,74]. 7KC and 24S induce a decrease in cell proliferation, assessed by crystal violet; an alteration of mitochondrial activity, quantified with the MTT test; increased permeability of plasma membrane, revealed with propidium iodide; overproduction of reactive oxygen species, revealed by dihydroethidium staining; caspase-3 cleavage; and activation of LC3-I into LC3-II that are typical of oxiapoptophagy. In agreement with previous studies, this complex type of cell death induced by 7KC and 24S is attenuated by α-tocopherol [36,38,39,74].

Natural products are considered effective sources for the discovery of powerful and novel therapeutic agents, particularly dietary phytochemicals (polyphenols, terpenes, carotenoids, and others). As some of them are known to protect against oxidative stress, they could be effective in preventing 7KC- and 24S-induced side effects and in preventing and/or treating 7KC- and 24S-associated diseases. At the moment, various phytochemicals (fatty acids, polyphenols, phytosterols, and tocopherols) are able to counter the side effects of oxysterols in different age-related pathologies [68]. Some of these phytochemicals, either food extracts or pure compounds, are present in Mediterranean diet products, such as olive oil, fruits, and vegetables.

In this context, it was interesting (i) to establish the chemical profile of MTSO from different areas of Tunisia (Zaghouan, Bizerte and Sousse) in phytosterols, tocopherols, and polyphenols, since these compounds are potentially neuroprotective, (ii) to determine their antioxidant characteristics, and (iii) to precisely determine their cytoprotective effects on nerve cells.

Currently, oxidative stress and mitochondrial dysfunction are considered key events in several degenerative diseases [77]. Interestingly, the chemical profile of MTSO reveals the presence of numerous compounds that can prevent oxidative stress and mitochondrial dysfunction. Thus, MTSO contains high levels of antioxidant molecules (tocopherols, polyphenols) capable of reducing the overproduction of reactive oxygen species (ROS: ROO•, RO•) [78], which can induce lipid peroxidation leading to protein carbonylation and several types of cellular dysfunction that may contribute to neurodegeneration [79,80]. The presence of these compounds is consistent with the antioxidant properties of MTSO determined with the KRL, DPPH, and FRAP tests. In addition, MTSO is also rich in oleic acid (C18:1 n-9). Although (C18:1 n-9) is not an antioxidant [47], it has been shown in 7KC-treated murine microglia BV-2 cells that this fatty acid is able to attenuate the overproduction of ROS [43,48]. In addition, oleic acid as well as α- and γ-tocopherol have been shown to prevent mitochondrial, lysosomal, and peroxisomal dysfunction in different types of nerve cells [33,34,43,48]. As these compounds, which have been shown to inhibit 7KC- and 24S-induced neurotoxicity are present at low levels in MTSO (the final concentrations of oleic acid, and α- and γ-tocopherol were more than one hundred-fold lower than those in the culture medium when these compounds were used alone), it is possible that some of them may act in synergy to exert cytoprotective effects and/or that several other compounds of MTSO (polyphenolic compounds, sylimarine, etc.), could be involved in the beneficial effects. We must also consider that the different compounds present in MTSO could activate and suppress several signaling pathways contributing to cytoprotection.

It should be noted that when 7KC was used at 25 and 50 μM, two concentrations capable of inducing oxidative stress and favoring oxiapoptophagy [38,80,81], MTSO was able to attenuate the toxicity induced by 7KC (25–50 μM). As observed with α- and γ-tocopherol [47], docosahexahenoic acid [38], acid oleic [46] and dimethylfumarate [72], and argan oil [47], MTSO (0.1% *v*/*v*) was able to counteract the inhibition of cell growth associated with 7KC-induced loss of cell adhesion.

These results were highlighted by the crystal violet and the MTT assays. As observed with α- and γ-tocopherol [46,47], docosahexahenoic acid [39], oleic acid [46], polyphenols [82], and dimethylfumarate [72], MTSO also significantly reduced the oxidative stress revealed by DHE staining as well as the percentage of cells permeable to PI. This increased the values of PI positive cells either indicates an increase of dead cells and/or an increase of cells with damaged plasma membranes resulting from lipid peroxidation which could be the consequence of ROS overproduction [4,43]. These cytoprotective effects of MTSO on the plasma membrane evocate those observed with argan oil which protects against the cytotoxic effects induced by 7KC [47]. In addition, 7KC- and 24S-induced mitochondrial dysfunctions were attenuated by MTSO—the mitochondrial dysfunction revealed by the MTT test was attenuated. In addition, MTSO was able to prevent 7KC-induced LC3-I activation in LC3-II and the cleavage of caspase-3, which are the criteria for autophagy and apoptosis, respectively. Currently, the autophagic process associated with 7KC-induced cell death is considered rather beneficial [83,84]. Since some compounds present in MTSO can normalize autophagy and attenuate apoptosis, which are two events involved in neurodegeneration, this is an important argument in favor of the potentially neuroprotective activity of MTSO.

So, it can be considered that MTSO is a mixture of compounds (fatty acids, polyphenols, tocopherols, and phytosterols) that can act synergistically. Polyphenols are mainly hydrophilic molecules present at very low levels in oil; their cytoprotective activity on 7KC-induced oxiapoptophagy can be excluded. On the other hand, fatty acids (especially oleic acid) and tocopherols (mainly α- and γ-tocopherol) are present at high concentrations, and it has already been proved that they prevent 7KC-induced oxiapoptophagy [34,48,72]. It is, therefore, supposed that these compounds probably contribute to the cytoprotective effects of MTSO. Since several data are available on the signaling pathways activated by 7KC [85] and on the cellular targets of tocopherols and oleic acid involved in the attenuation of 7KC-induced side effects [43,86], it will be easy to determine whether this assumption is realistic. 

It is noteworthy that in the presence of nigella seed oil, considered potentially toxic, major differences compared to MTSO are observed. Whereas MTSO is cytoprotective, nigella seed oil is not; it is cytotoxic and does not prevent 7KC- and 24S-induced side effects. In vivo, toxic effects of nigella seed oil have also been reported by Zaoui et al. [87], who showed that certain blood parameters were modified, especially the platelet count. Other more recent in vivo studies by Zaghloul et al. [88] confirmed the toxic effect of nigella seed oil on the renal cortex and, to a lesser degree, on hepatic cells. In addition, whereas the content of fatty acids is almost similar in MTSO and nigella seed oil, there are major differences in their sterol contents. It is suggested that, the cytotoxicity of nigella seed oil could be explained, at least in part, by the high content of cycloartenol and methylene cycloartenol which are not found in MTSO. Thymoquinone, which is a polyphenol known for its anti-inflammatory, antioxidant, and anti-proliferative properties, is also only found in MTSO, but its concentration is probably too low to induce side effects [89]. 

Altogether our data support that our in vitro assay on 158N cells, which is rapid and easy to standardize, could be used as a screening test in order to distinguish and identify oils with different biological activities. 

In addition, the present study provides new data that reinforce the interest in the use of MTSO for the prevention of neurodegenerative diseases. The chemical profile of MTSO was quite similar in the three areas of Tunisia studied (Bizerte, Zaghouan, and Sousse), and revealed the presence of fatty acids, polyphenols, phytosterols, and tocopherols capable of attenuating the side effects associated with neurodegeneration (oxidative stress, mitochondrial dysfunction, and apoptosis/autophagy). These chemical characteristics reinforce the interest in the potential of MTSO to prevent age-related diseases including neurodegenerative diseases, since several of its compounds are theoretically capable of crossing the blood–brain barrier and have been shown to have neurotrophic (cytoprotective + differentiating) properties on nerve cells, mainly oleic acid and polyphenols [90,91]. In addition, MTSO has antioxidant properties and is also capable of counteracting 7KC- and 24S-induced oxiapoptophagy in 158N murine oligodendrocytes. Since 7KC and 24S can be increased in patients with neurodegenerative diseases, the cytoprotective effects of MTSO against the toxic effects of 7KC and 24S suggest that MTSO may have beneficial effects related to preventing or slowing down the development of 7KC- and 24S- associated neurodegenerative diseases. In addition, the results obtained on the cytoprotective effects of milk thistle seed oil on 158N cells encourage us to develop more elaborate cell models that take into account the selective passage of certain oil compounds through the blood–brain barrier. Models mimicking the blood–brain barrier by combining endothelial cells, pericytes, and nerve cells [92,93,94] should provide a more accurate evaluation of the activity of MTSO on brain cells under normal and pathologic conditions induced by mechanical, physical and/or chemical agents.

## 4. Conclusions

Based on the biochemical and antioxidant characteristics of MTSO, this oil may be beneficial for age-related diseases, including neurodegenerative diseases, and may be associated with diet as functional foods. Indeed, these oils contain numerous molecules (fatty acids, polyphenols, phytosterols, and tocopherols) that are theoretically capable of crossing the blood–brain barrier [92,93,94] and attenuating numerous neurodegenerative side effects: oxidative stress, mitochondrial dysfunctions, and cell death [36,37,39,46]. In addition, the various compounds present in the MTSO could act in synergy to counter the toxic effects of 7KC of 24S identified at increased levels in patients with neurodegenerative diseases. The data shown in this study also validate the development of a cellular model (158N cells treated with oxysterols) to apprehend the biological activities of oils on brain cells.

## Figures and Tables

**Table 1:** Saturated fatty acid and unsaturated fatty acid contents expressed as percentage of total fatty acids (FA) in milk thistle seed oil (MTSO) and nigella seed oil (NSO).

Fatty Acids		Origins of Milk Thistle Seed Oil (MTSO)		Nigella Seed Oil (NSO)
	Sousse	Zaghouan	Bizerte	
C12:0	0.00	0.00	0.11 ± 0.00	0.00
C14:0	0.08 ± 0.00	0.07 ± 0.02	0.13 ± 0.00	0.16 ± 0.001
C15:0	0.00	0.00	0.00	0.00
C16:0	13.06 ± 0.07	6.25 ± 2.00	8.89 ± 0.14	12.29 ± 0.043
C17:0	0.09 ± 0.00	0.08 ± 0.03	0.10 ± 0.00	0.07 ± 0.00
C18:0	3.35 ± 0.11	4.03 ± 1.32	5.21 ± 0.02	3.37 ± 0.05
C20:0	1.48 ± 0.06	2.33 ± 0.71	2.72 ± 0.09	0.22 ± 0.01
C22:0	0.76 ± 0.00	1.86 ± 0.59	1.99 ± 0.11	0.05 ± 0.00
C24:0	0.19 ± 0.00	0.40 ± 0.13	0.46 ± 0.03	0.00
C16:1 n-7	19.24 ± 0.17	18.98 ± 0.80	19.63 ± 0.10	0.22 ± 0.00
C16:1 n-9	0.00	0.00	0.00	0.00
C18:1n-9	21.39 ± 0.02	15.78 ± 4.95	19.03 ± 0.01	22.76 ± 0.02
C18:1 n-7	0.79 ± 0.01	0.40 ± 0.13	0.57 ± 0.01	1.09 ± 0.00
C18:2 n-6	56.77 ± 0.57	48.70 ± 14.94	58.47 ± 0.20	54.56 ± 0.14
C20:1 n-9	0.31 ± 0.00	0.72 ± 0.21	0.81 ± 0.01	0.32 ± 0.00
C18:3 n-3	0.49 ± 0.01	0.11 ± 0.03	0.16 ± 0.00	1.54 ± 0.02
C20:2 n-6	0.00	0.00	0.00	2.25 ± 0.02
C22:1 n-9	0.00	0.06 ± 0.04	0.00	0.11 ± 0.02
∑SFA	19.24 ± 0.17	18.98 ± 0.80	19.63 ± 0.10	16.18 ± 0.01
∑UFA	80.83 ± 1.86	83.30 ± 4.39	79.12 ± 0.20	82.85 ± 0.14

SFA: saturated fatty acids; UFA: unsaturated fatty acids. Values are the mean ± SD of three determinations.

**Table 2:** Tocopherol content (mg kg^−1^) of milk thistle seed oil (MTSO).

Area from Tunisia	Bizerte	Zaghouan	Sousse
Tocopherol			
α	47.65 ± 3.54	286.22 ± 25.49	278.47 ± 24.64
β	1.91 ± 0.21	3.58 ± 0.37	6.66 ± 0.74
γ	0.0	14.24 ± 1.25	23.94 ± 2.14
δ	0.0	14.24 ± 1.22	5.23 ± 0.61
Total	49.57 ± 5.11	318.29 ± 28.45	314.31 ± 30.77

Values are the mean ± SD of three determinations.

**Table 3:** Sterol and phytosterol contents (% of total sterols) of milk thistle seed oil (MTSO) and nigella seed oil (NSO).

Sterols		Origins of Milk Thistle Seed Oil (MTSO)		Nigella Seed Oil (NSO)
	Bizerte	Zaghouan	Sousse	
Cholesterol	11.47 ± 0.04	4.53 ± 0.01	9.53 ± 0.03	0.55 ± 0.01
Campesterol	4.75 ± 0.01	4.77 ± 0.02	10.89 ± 0.03	6.97 ± 0.06
∆7-Campesterol	4.14 ± 0.01	4.81 ± 0.02	2.73 ± 0.02	0.18 ± 0.03
Spinasterol	0	0	0	0
β-Sitosterol	31.96 ± 0.13	32.78 ± 0.05	42.33 ± 0.01	34.91 ± 0.30
Schotenol	20.97 ± 0.09	24.54 ± 0.12	6.86 ± 0.03	0.51 ± 0.03
Stigmasterol	20.97 ± 0.09	5.91 ± 0.03	4.88 ± 0.01	6.92 ± 0.09
β-amyrine	4.72 ± 0.01	5.14 ± 0.04	3.08 ± 0.01	1.29 ± 0.08
∆5 avenasterol	2.83 ± 0.03	3.03 ± 0.02	4.59 ± 0.02	8.50 ± 0.05
cycloartenol	2.14 ± 0.07	1.67 ± 0.03	1.51 ± 0.05	18.34 ± 0.21
∆7 avenasterol	3.82 ± 0.05	4.68 ± 0.06	3.98 ± 0.07	1.33 ± 0.06
24-Methylene cycloartenol	2.07 ± 0.03	2.40 ± 0.02	2.52 ± 0.01	12.55 ± 0.10
24-Methylene cholesterol	0.25 ± 0.01	0.20 ± 0.01	0.77 ± 0.00	0.82 ± 0.03
Campestanol	0.50 ± 0.01	0.54 ± 0.02	0.91 ± 0.02	0.79 ± 0.01
∆7-Stigmasterol	0	0	0	0
Clerosterol	0	0	0	0.85 ±0.05
Graminasterol	1.31 ± 0.04	1.54 ± 0.06	1.39 ± 0.02	1.80 ± 0.04
Lupeol	0	0	0	0
Fucosterol	1.77 ± 0.01	2.03 ± 0.05	2.79 ± 0.00	0.91 ± 0.12
Citrostadienol	1.57 ±0.01	1.43 ± 0.00	1.24 ± 0.00	2.78 ± 0.11
Total content (mg/kg)	5206.13 ± 24.23	5088.54 ± 71.96	5891.82 ± 118.12	2659.29 ± 189.56

Values are the mean ± SD of three determinations.

**Table 4:** Polyphenol contents (mg equivalents quercetin/100 g of oil) of milk thistle seed oil (MTSO) and nigella seed oil (NSO).

Polyphenols		Origins of Milk Thistle Seed Oil (MTSO)		Nigella Seed Oil (NSO)
	Bizerte	Zaghouan	Sousse	
Homovanillic acid	ND	0.13	ND	0.19
Vanillin	ND	0.20	0.33	0.23
p-Coumaric acid	ND	0.07	ND	ND
Quercetine-3β-glucoside	ND	0.08	ND	ND
Quercetin	ND	0.12	ND	0.13
Apigenin	ND	0.09	ND	ND
2,6-Dihydroxybenzoïc acid	ND	ND	ND	1.27
Chlorogenic acid	ND	ND	ND	0.09
Ferrulic acid	ND	ND	ND	0.09
Thymoquinone	ND	ND	ND	0.70
Hydroxytyrosol	ND	ND	ND	ND
Tyrosol	ND	ND	ND	ND
Oleuropein	ND	ND	ND	ND
Luteoline	ND	ND	ND	ND
Protocatechic acid	ND	ND	ND	ND

ND: not detected. Values are the mean ± SD of three determinations.

**Table 5:** Estimation of the antioxidant activities of milk thistle seed oil (MTSO) with the KRL (Kit Radicaux Libres), the ferric reducing antioxidant power (FRAP) test, and the (DPPH) test.

Compounds	Antioxidant Activities (Trolox Equivalent)		
	KRL	FRAP	DPPH
α-Tocopherol	0.94 ± 0.01	0.80 ± 0.06	1.33 ± 0.03
Resveratrol	7.90 ± 0.05	5.27 ± 0.03	4.52 ± 0.04
Silymarin	3.67 ± 0.03	3.47± 0.07	2.96 ± 0.02
Ferrulic acid	4.73 ± 0.05	2.02 ± 0.04	3.36 ± 0.03
Zaghouan MTSO	144.25 ± 0.58	211.06 ± 0.42	216.16 ± 0.45
Bizerte MTSO	180.48 ± 0.49	125.122 ± 0.35	139.480 ± 0.53
Sousse MTSO	160.173 ± 0.33	122.14 ± 0.47	98.54 ± 0.29
Nigella seed oil	ND	312.29 ± 0.35	227.53 ± 0.28

Data are presented in Trolox equivalents for the references (α-tocopherol, resveratrol, silymarin, ferrulic acid): one mole of α-tocopherol, resveratrol, silymarin, ferrulic acid is equivalent to X moles (values shown in the table) of Trolox. For the MTSO from Zaghouan, Bizerte, and Sousse as well as for nigella seed oil, data are also presented in Trolox equivalents: 1 mL of oil is equivalent to X moles (values shown in the table) of Trolox. Data shown are the mean of three independent experiments conducted in triplicate. ND: not detected.

## Supplementary Materials

The following are available online at http://www.mdpi.com/2076-3921/7/7/95/s1. Figure S1: Nuclear morphology of normal and apoptotic cells after staining with Hoechst 33342.
