# Supplementary material for: Cytoprotective Activities of Milk Thistle Seed Oil Used in Traditional Tunisian Medicine on 7-Ketocholesterol and 24S-Hydroxycholesterol-Induced Toxicity on 158N Murine Oligodendrocytes
Source: Antioxidants (Basel). 2018 Jul 19;7(7):95. doi: 10.3390/antiox7070095 (PMC6071139; doi:10.3390/antiox7070095)
Supplement: Supplementary file 1 [file antioxidants-07-00095-s001.pdf]

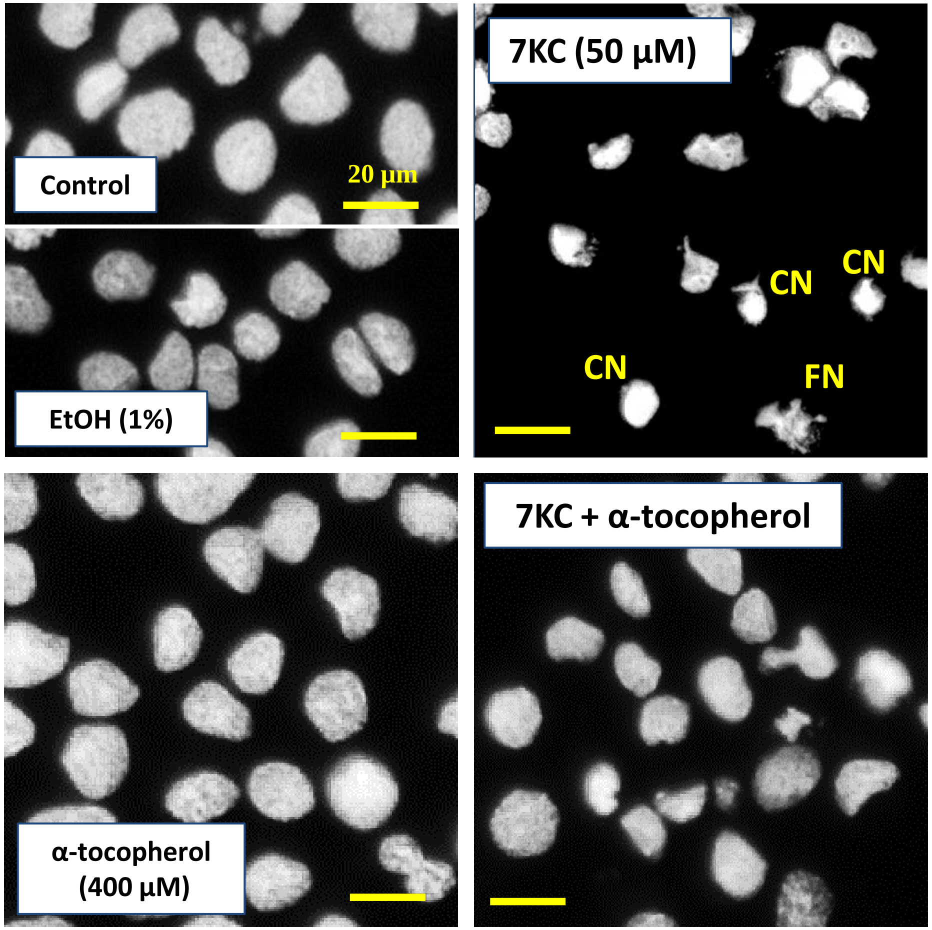

<u>Supplementary Fig. 1:</u> Nuclear morphology of normal and apoptotic cells after staining with Hoechst 33342. After 24 h of culture, 158N murine oligodendrocytes were cultured for 24 h without or with 7KC (50 µM) in the absence or presence of MTSO (Zaghouan; 0.1% v/v) or α-tocopherol (400 µM) used as positive control. Whereas cells with round and regular nuclei were observed in control, vehicle (EtOH)- and alpha-tocopherol-treated cells, several cells with condensed (CN) and fragmented nuclei (FN) were observed in the presence of 7KC (similar features were observed with 24S). When 7KC (or 24S) was associated with alpha-tocopherol, mainly cells with with round and regular nuclei were observed. In the presence of milk thistle seed oil, the effects were similar than with alpha-tocopherol. CN: condensed nuclei; FN: fragmented nuclei.
